# *Aedes aegypti* miRNA-33 modulates permethrin induced toxicity by regulating VGSC transcripts

**DOI:** 10.1038/s41598-021-86665-6

**Published:** 2021-03-31

**Authors:** Tristan D. Kubik, Trey K. Snell, Karla Saavedra-Rodriguez, Jeffrey Wilusz, John R. Anderson, Saul Lozano-Fuentes, William C. Black, Corey L. Campbell

**Affiliations:** Department of Microbiology, Immunology and Pathology, Colorado State University, Campus Delivery 1685, Fort Collins, CO 80523 USA

**Keywords:** Genetics, Eukaryote, Evolutionary genetics, Population genetics

## Abstract

*Aedes aegypti* is a major vector of Zika, dengue, and other arboviruses. Permethrin adulticidal spraying, which targets the voltage-gated sodium channel (VGSC), is commonly done to reduce local mosquito populations and protect humans from exposure to arbovirus pathogens transmitted by this dangerous pest. Permethrin resistance, however, is a growing problem and understanding its underlying molecular basis may identify avenues to combat it. We identified a single G:C polymorphism in pre-miR-33 that was genetically associated with permethrin resistance; resulting isoforms had structural differences that may affect DICER-1/pre-miRNA processing rates. We then assessed the effects of overexpression of pre-miR-33 isoforms on permethrin toxicological phenotypes, VGSC transcript abundance and protein levels for two genetically related mosquito strains. One strain had its naturally high permethrin resistance levels maintained by periodic treatment, and the other was released from selection. VGSC protein levels were lower in the permethrin resistant strain than in the related permethrin-susceptible strain. Overexpression of the G-pre-miR-33 isoform reduced VGSC expression levels in both strains. To further elucidate changes in gene expression associated with permethrin resistance, exome-capture gDNA deep sequencing, genetic association mapping and subsequent gene set enrichment analysis revealed that transport genes, in particular, were selected in resistant versus susceptible mosquitoes. Collectively, these data indicate that miR-33 regulates VGSC expression as part of a nuanced system of neuronal regulation that contributes to a network of heritable features determining permethrin resistance.

## Introduction

The RNA interference (RNAi) pathway relies on protein/microRNA (miRNA) complexes to regulate gene expression in development, cellular homeostasis and in response to stress. RNA-induced silencing complexes (miRISCs) bind to mature miRNAs and subsequently inhibit target protein translation through one of several mechanisms (reviewed in^1^). In one mechanism, miRNA RNA interference (miRNAi) acts through the RNA decay pathway^2–4^. In mosquitoes, as in all dipterans, ARGONAUTE-1 (AGO1) and DICER-1 are critical members of the miRISC^5–7^ and work together to process pre-miRNAs^8^. AGO1-miRNA binding determines which mRNAs are targeted for translational repression or destabilization^9^. Thus, polymorphisms in pre-miRNAs could alter protein binding and subsequently affect the rate of pre-miRNA processing in vivo^10,11^.

The voltage-gated sodium channel (VGSC), a motoneuronal transport protein, is the target of all pyrethroids and DDT (dichlorodiphenyltrichloroethane) in *Ae. aegypti*^12^. Binding of permethrin to VGSC causes paralysis and is fatal to susceptible mosquitoes, whereas resistant mosquitoes remain unaffected or quickly recover from the typical toxicological response, also known as knockdown^13^. The mechanism of permethrin toxicity has been well-described through the analysis of target site mutations (KDR) and metabolic resistance^13–23^; moreover at least two permethrin binding sites are present on VGSC molecules^12^. In *Drosophila* spp., VGSC translation is controlled by the RNA decay effector PUMILIO^24,25^. However, the molecular mechanisms underlying VGSC translational control have not been investigated in mosquitoes. Because VGSC is the direct target of permethrin, regulation of VGSC protein levels could condition the toxicological response.

In previous work, we identified the genetic association of neural synapse related proteins, including VGSC*,* to pyrethroid resistance in *Ae. aegypti*^26^. For the current work, we further interrogated published genetic association data^26^ and identified a polymorphism in pre-miRNA miR-33, consistent with a possible role in knockdown or metabolic resistance. In drosophilids, miR-33 regulates fatty acid metabolism and gonadogenesis^27,28^; the miR-33 gene resides in the intron of sterol-regulatory binding protein 1 (SREBP1*,* AAEL024184), which regulates lipid metabolism^29^. Fatty acid oxidation is regulated through the miR-33 target, mitochondrial carnitine palmitoyltransferase I (CPT1)^27,30^. In miR-33 over-expressing drosophilid transgenic lines, triacylglycerides (TAGs) and other neutral fatty acids accumulate upon starvation, a type of AKT (protein kinase B)/insulin signaling pathway-induced stress response^27^.

Here, we explored miR-33’s role in permethrin resistance by determining the effects of miR-33 over-expression and depletion on toxicological phenotypes of paralysis, or knockdown, and lethality using insecticide bottle assays. VGSC transcript and protein levels were evaluated in two mosquito strains derived from the same genetic background. High throughput exome-capture deep sequencing was performed on mosquito pools for genetic association analysis^26,31,32^ of features under selection by permethrin treatment. We found that miR-33 modulates VGSC transcript and protein levels. Moreover, strain-specific differences in single nucleotide polymorphisms (SNPs) in genes in a variety of functional categories were associated with permethrin susceptibility. Collectively, these data indicate that miR-33, as a major regulator of VGSC expression, is part of a network of mechanisms, which include VGSC genotype, metabolic resistance and other heritable features of resistance.

## Results

### Pre-miR-33 polymorphism has genetic association to permethrin resistance

We determined the extent of predicted miRNA coverage in our previously published genomic DNA (gDNA) deep sequencing datasets^26,32^, and found that seven miRNAs were present at sufficient coverage for genetic association analysis (Supplementary Fig. S1). The datasets were interrogated to identify SNPs in pre-miRNAs with χ^2^ contingency − log_10_(χ^2^ *p* value) scores greater than 3.0 (Supplementary Table S1). A single polymorphism was identified; this G:C transition site was at position # 50888906 in chromosome 1 of the Viva Caucel collection (see “Methods”), within the pre-miRNA of miR-33 and the contiguous intron 3–4 of SREBP1.

### Pre-miR-33 structure analysis via nuclease sensitivities

**Figure 1:**
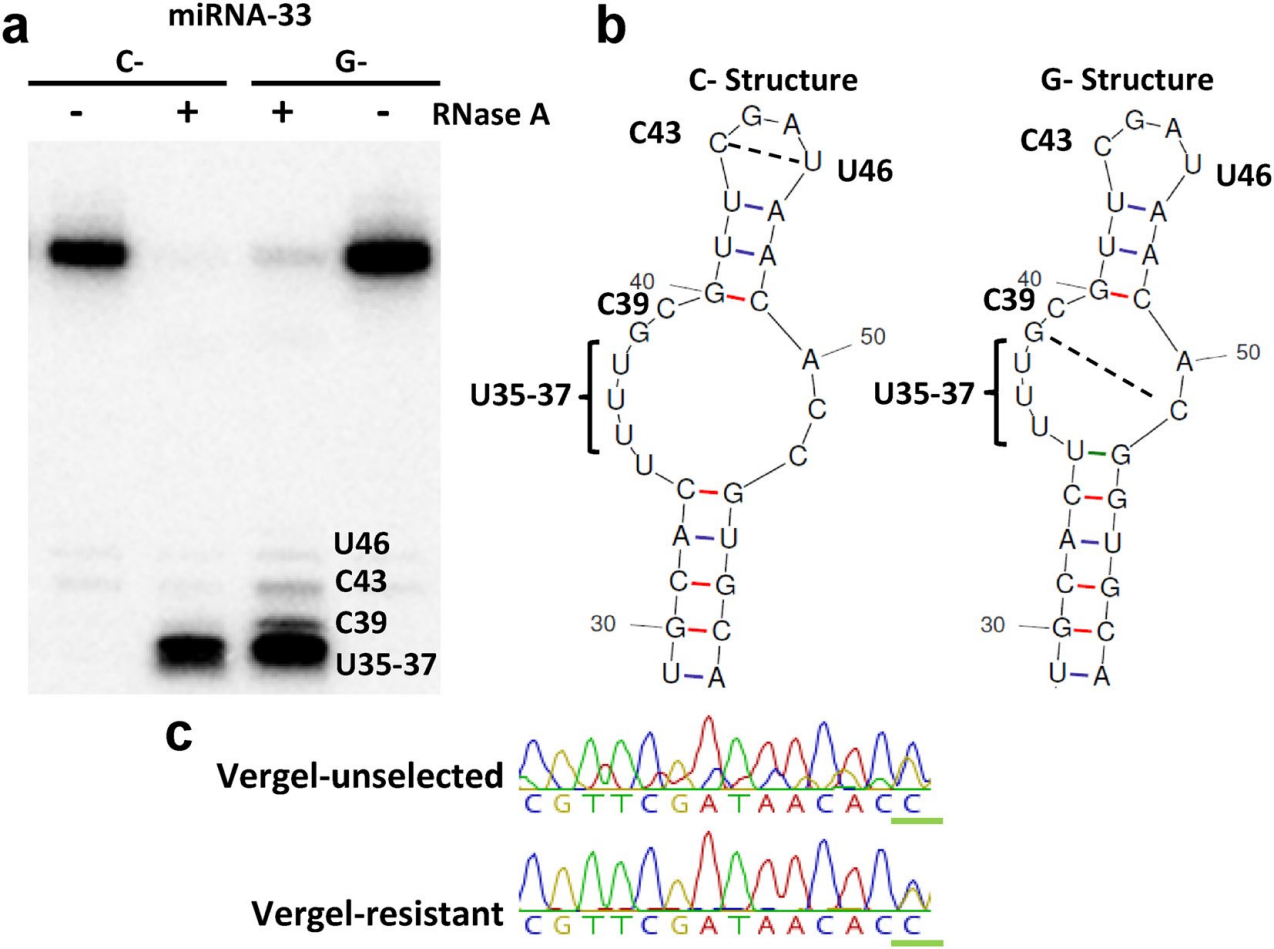
Secondary structures for pre-miR-33 hairpins were altered in the presence of a single nucleotide polymorphism. (**a**) RNAse A treatment of C-pre-miR-33 and G-pre-miR-33. Nucleotide positions were assigned using an RNA ladder as well as RNA partially digested using T1 RNAse to identify the positions of G residues. Data are representative of at least 3 technical replicates. (**b**) Alternative secondary structures with cleavable single-stranded RNA position numbers are noted. (**c**) Sanger sequencing of the pre-miRNA region of pools of 5 mosquitoes from each strain (Vergel-unselected (VU) and Vergel-resistant (VR)); G:C polymorphism is underlined in green. Raw blot is in Supplementary Fig. S2. Chromatograms were produced in Geneious (version 11.1 (Geneious.com)), with permission.

The polymorphism of interest was located adjacent to the mature miRNA, such that the secondary structure of the pre-miRNA hairpin showed alternative isoform-dependent conformations (Fig. 1). Interestingly, the SNP was also near the expected site for DICER-1 maturational cleavage, required to form mature miRNAs^33^. Changes to pre-miRNA secondary structure can alter miRNA processing rates^10,11^. However, once processed, regulatory function of the resulting mature miRNAs should remain unaffected. Therefore, we hypothesized that miR-33 is involved in permethrin resistance, and subsequent differences in miRNA processing rates, induced by C- and G-miR-33 overexpression, could affect permethrin toxicological phenotypes. In heterozygotes, differential expression from each isoform could condition target mRNA regulation rates.

The free energy (ΔG) of folding for G-pre-miR-33 was − 31.70, whereas the ΔG for the C-isoform was − 35.30 (mfold^34^), indicating higher stability of the C-isoform. Structural differences were also detected between C- and G-pre-miR-33 RNAs in vitro. Nuclease mapping with single-strand-specific RNase A revealed that G-pre-miR-33 had increased sensitivity at three positions relative to the C-isoform, consistent with the presence of single-stranded pyrimidine residues that were not present in the C-isoform (Fig. 1a). This suggested that the structure of the top portion of the G-isoform RNA likely is more breathable than the C-isoform (Fig. 1b). More conformational flexibility would be expected to facilitate binding by processing or regulatory proteins, such as DICER-1 ribonuclease and AGO1, which work together in miRNA maturation and loading into the miRISC^35^, respectively. Specifically, alterations in miRNA loop size affect loading by AGO1^10^, a major component of miRNAi.

### Establishment of permethrin resistant and susceptible colonies

**Figure 2:**
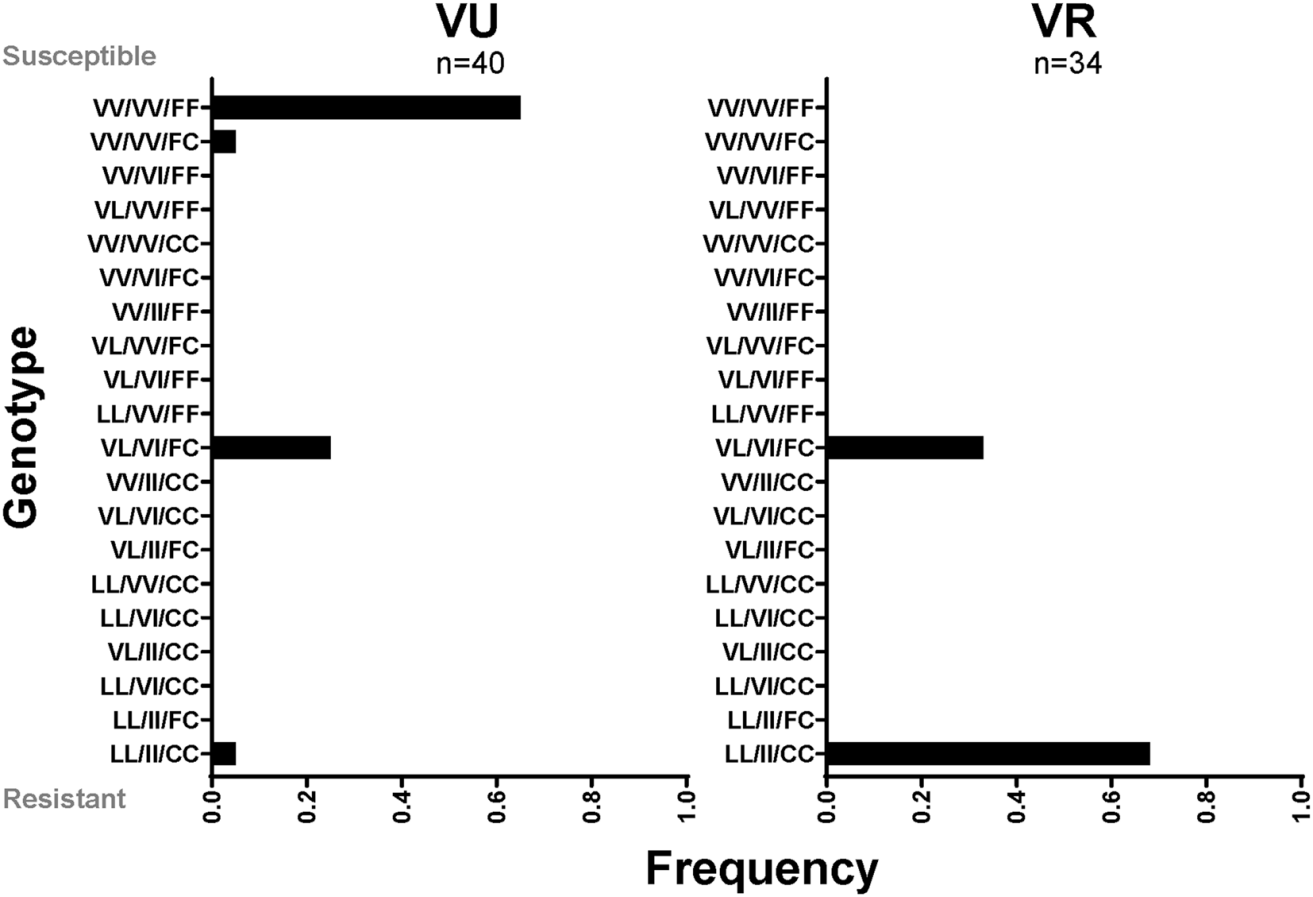
KDR genotypes present in VU and VR. Resistant alleles are V410L, V1016I, F1534C; the order of genotypes is 410, 1016, 1534. VV/VV/FF is indicative of a complete absence of KDR alleles (homozygous susceptible), LL/II/CC is homozygous resistant and VL/VI/FC represents heterozygosity at all 3 alleles (Prism GraphPad version 8.0., https://www.graphpad.com/scientific-software/prism/).

Because the Viva Caucel collection was not maintained in colony, subsequent analyses were performed using Vergel mosquitoes (generations F_24_–F_27_). Two mosquito colonies arose from the original field collection (Vergel, Yucatan, 2012)^36^; one was exposed every 3rd generation to permethrin (VR, Vergel-resistant), whereas the other (VU, Vergel-unselected) was not. For this reason, VU lost permethrin resistance over time. In bottle assays, the permethrin LC_50_ (lethal concentration 50%) for the unselected Vergel strain (VU) was ~ 2.4 μg (Table 1), whereas the LC_50_ for the VR strain was ~ 25 μg, which is similar to that of the founding collection^36,37^. Resistance ratios of VR were about 10 times than that of VU (Table 1). VGSC KDR allele frequencies were also calculated for leucine 410 (V410L), isoleucine 1016 (V1016I, housefly annotation) and cysteine 1534 (F1534C). As expected, the VR strain had a high proportion of homozygous resistance KDR genotypes (67%) (Fig. 2). Notably, VU also retained resistance alleles; specifically, 25% of individuals assayed maintained heterozygous genotypes at all three alleles.

**Table 1:** Resistant vs susceptible strains—LC_50_ for permethrin treatment.

Lethal concentration 50%	New orleans	VU strain, F_27_	VR strain, F_27_	Resistance ratio VR/VU
LC_50_	0.63	2.41	24.92	10.35
Lower cI*	0.56	2.27	23.24	
Upper cI	0.72	2.56	26.72	

*cI, confidence interval of µg permethrin treatment.

### Pre-miR-33 allele frequencies are similar in VR and VU

Female mosquito pools of both strains carry both G- and C-miR-33 alleles (Fig. 1c). To determine the allele frequency of C- and G-pre-miR-33 isoforms in individual mosquitoes, we performed genotyping and Sanger sequencing on selected individuals to detect C- or G-SNPs. Results indicated allele frequencies were not different among the two colonies (Supplementary Table S2). Therefore, while pre-miR-33 allele differences may contribute to the resistance phenotype in the Viva Caucel collection, we conclude that altered processing of the pre-miR-33 allele is not a key contributor to permethrin resistance in the Vergel strain. Given the strong selective pressure of pyrethroids, it is perhaps not surprising that multiple pathways of resistance can arise. We thus turned our attention to deciphering additional genetic factors involved in resistance.

### Pre-miR-33 polymorphism alters mature miR-33 levels in VU mosquitoes

**Figure 3:**
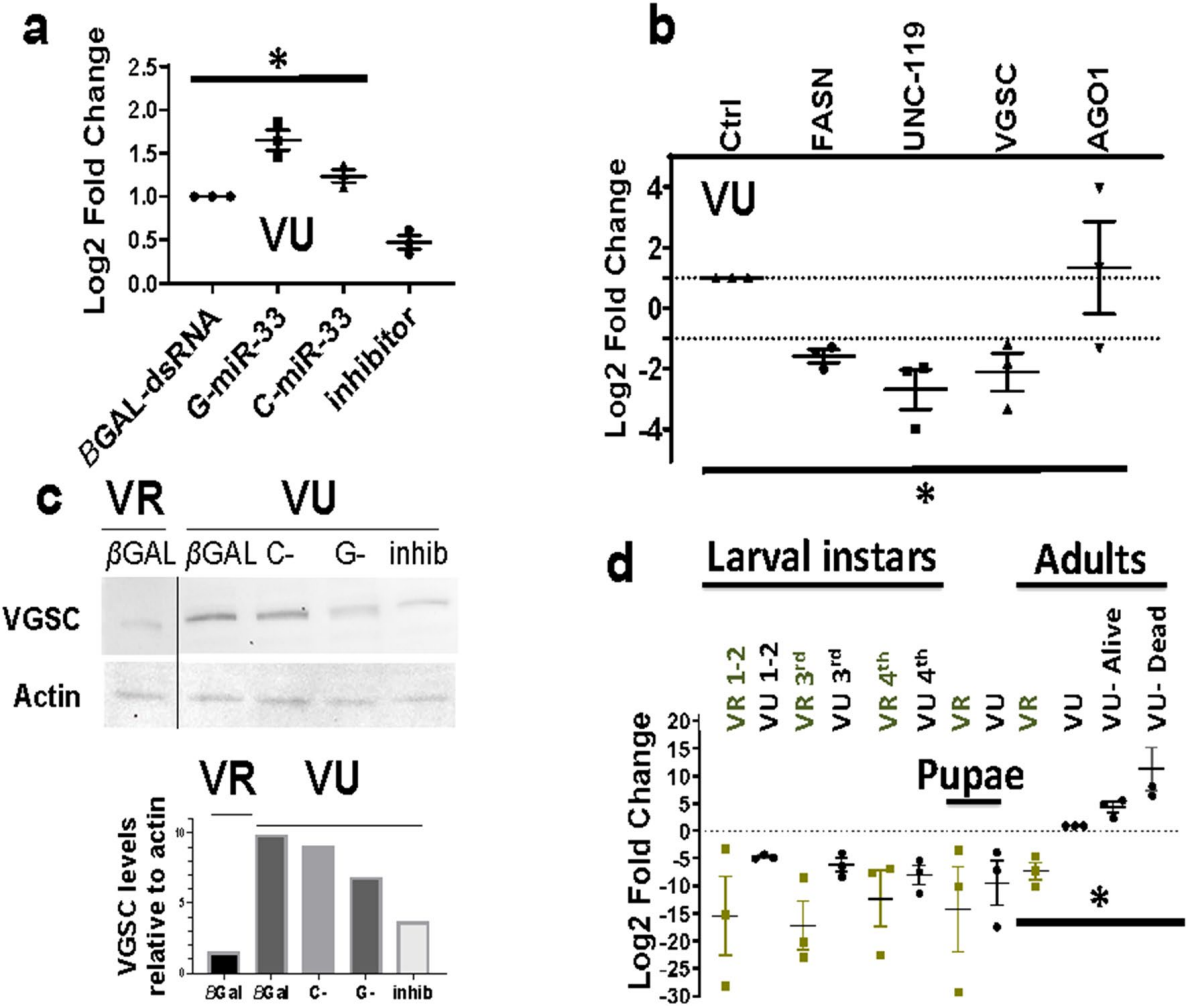
VGSC transcript and protein levels following miR-33 isoform over-expression and at different life stages in VU and VR. (**a**) VU (Vergel-unselected) miR-33 levels were measured by RT-qPCR at 3 days post-treatment (dpt) relative to let-7 miRNA reference standard and compared to endogenous levels in βGAL-injected controls (ANOVA, *p = 0.043). (**b**) Decay-sensitive transcripts resulting from G-pre-miR-33 over-expression in VU (ANOVA, *p = 0.015), relative to ACTIN and ribosomal protein S7 (RPS7) reference standards and βGAL-dsRNA injected controls (Ctrl). Targets- FASN- fatty acid synthase, UNC-119 GMP phosphodiesterase, VGSC- voltage-gated sodium channel, ARGONAUTE1 (AGO1), component of the miRISC. Error bars indicate mean with standard error of the mean (SEM). (**c**) Representative immunoblots of VGSC in VU following G-pre-miR-33 or C-pre-miR-33 pre-miRNA over-expression or treatment with a miR-33 depletion oligo (inhib) and VR controls. Protein was extracted from pools of 5 mosquitoes, and equivalent amounts of protein were loaded per lane. Top panel, 200 µg protein per lane; bottom panel, histogram of relative band intensities. Apparent size of VGSC protein, 100 kDa. Raw blots are shown in Supplementary Fig. S4. Data is a representative example from 3 biological replicates. (**d**) Relative VGSC transcript levels throughout the life stages were measured relative to RPS7 and ACTIN, using adult female VU (Vergel-unselected) as the calibrator. One VU subpopulation exhibited the knockdown resistant phenotype upon permethrin exposure (VU-Alive) and the other was comprised of pooled mosquitoes that died following challenge (VU-Dead) (one-way ANOVA, *p = 0.038). Pools of 3 adult female mosquitoes were used for each group; pools of (20–50) immature life stages were collected. Data represents compilation of 3 biological replicates. Error bars indicate mean with SEM (Prism GraphPad version 8.0., https://www.graphpad.com/scientific-software/prism/).

To test whether processing rates differed in vivo among G- or C-pre-miR-33 isoform treated pools, adult VU and VR mosquitoes were injected with in vitro-transcribed pre-miRNAs, and mature miRNA levels were tested by reverse transcription quantitative PCR (RT-qPCR). At 3 days post- G-pre-miRNA treatment (dpt), mature miR-33 levels were significantly higher than in control groups in VU (Fig. 3a, ANOVA, *p = 0.043), whereas, in VR, neither isoform was associated with miRNA levels significantly different from endogenous levels (Supplementary Fig. S3a). Treatment with a sequence-specific miRNA inhibitor reduced miR-33 levels, as expected, in both treatment groups (Fig. 3a, Supplementary Fig. S3a). The lack of complete miR-33 ablation by the inhibitor was likely due to incomplete penetrance of the injected inhibitor into all tissues.

### MiR-33 targets include VGSC

PUMILIO is an RNA decay effector that is responsible for regulation of PARA (VGSC) transcripts in *Drosophila*^38^ and, thus, regulates neural synapse function^25^. PUMILIO was identified in our previous genetic association study for discovery of genes associated with pyrethroid resistance^26^. Further, RNA decay is known to act in concert with miRNAi to control gene expression (reviewed in^1^). VGSC and predicted miR-33 targets from our earlier studies^26,39^ were interrogated to identify those regulated by miRNAi RNA decay. Upon screening VU, VGSC transcripts were significantly reduced in the presence of excess G-pre-miR-33 relative to controls (Fig. 3b), consistent with miRNAi-induced decay. All transcripts tested in VR, including VGSC, were depleted in the presence of excess G-pre-miR-33 (Supplementary Fig. S3b).These results are consistent with the hypothesis that miR-33 regulates VGSC expression levels, either directly or indirectly, in concert with PUMILIO in *Ae. aegypti.* Basal VGSC protein levels (βGAL-injected) were lower in VR than in VU (Fig. 3c). Moreover, in VU, G-pre-miR-33 over-expression led to lower VGSC protein levels than for analogous C-pre-miR-33-injected or control mosquitoes, as expected if pre-miRNA processing rates were isoform-specific.

### Low VGSC levels are associated with resistance phenotypes

VGSC gene expression patterns were defined throughout the life stages for VU, VR and following permethrin treatment relative to untreated adult female VU mosquitoes (Fig. 3d). VGSC transcripts were evident in all life stages at low levels in VR relative to untreated VU adult females. Consistent with VGSC protein levels in controls, adult VR VGSC transcript levels were significantly lower than in VU adults (Fig. 3d, ANOVA, *p = 0.038). Consistent with this, basal miR-33 levels were significantly higher in VR over VU adults (Supplementary Fig. S3c). Among permethrin-treated VU adults at 3 dpt, knockdown-resistant pools showed a trend toward lower VGSC transcript levels than did adults that died by 3 dpt (Fig. 3d). In addition, permethrin-treated VU mosquitoes showed a trend toward increased VGSC levels compared to untreated adult VU, consistent with transcriptional stimulation following permethrin treatment.

### MiR-33 overexpression reduced permethrin-induced mortality in Vergel mosquitoes

**Figure 4:**
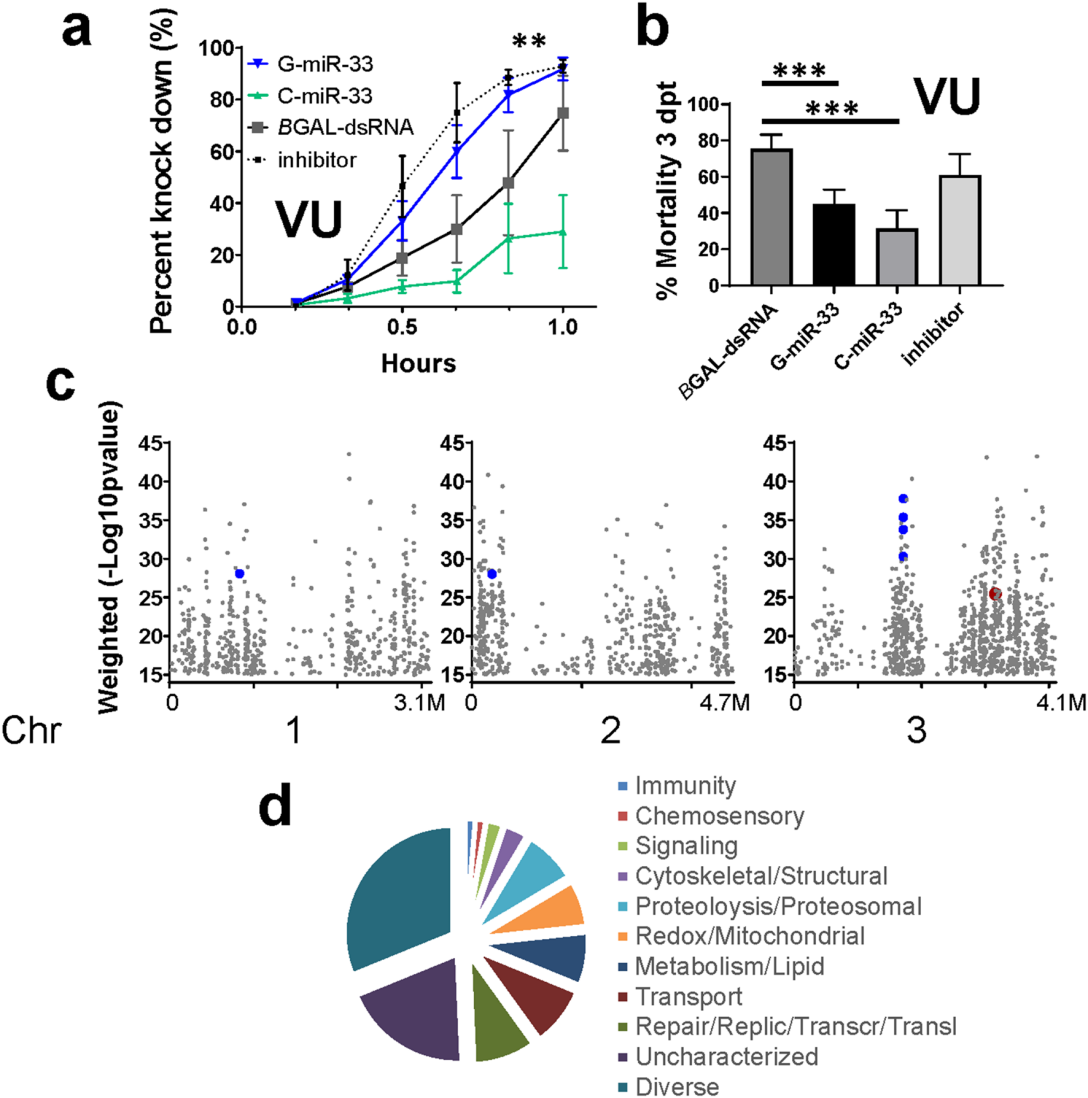
VU permethrin toxicological response in the presence of excess miR-33 isoforms and VR vs VU strain genetic association data. (**a**) MiR-33 pre-miRNA over-expression affects permethrin toxicological response (knockdown) in VU. G-pre-miR-33 or C-pre-miR-33 were over-expressed in VU (Vergel-unselected) adult mosquitoes, or depleted with inhibitor, followed by standard bottle assays using a discriminating permethrin dose (1.5 µg per bottle) (ANOVA **p = 0.0042). (**b**) Mortality of individuals from treatment in (**a**) at 3 days post permethrin exposure (Fisher’s Exact test, G-***p = 0.0002 and C-***p = 1.0 × 10^–5^). (**c**) Genetic association analysis of target-capture gDNA deep sequencing shows genes arranged according to physical location along chromosomes. VGSC, − log_10_ (χ^2^ *p* value) 25.43 indicated in red, CYPs with scores higher than VGSC are highlighted in blue and enlarged for emphasis (Supplementary Table S3). (**d**) Two hundred ninety two genes with − log_10_ (χ^2^ *p* value) higher than VGSC were categorized according to predicted functional groups. Functional categories are indicated clockwise from top. Immunity 1.3%, Chemosensory 1.3%, Signaling 2.4%, Cytoskeletal/Structural 3.4%, Proteolysis/Proteosomal 7.9%, Redox/Mitochondrial 6.9%, Metabolism/Lipid 7.9%, Transport 8.9%, Repair/DNA Replication/Transcription/Translation (RRTT) 9.3%, Uncharacterized 19.5%, Diverse 31.1% (Prism GraphPad version 8.0., https://www.graphpad.com/scientific-software/prism/).

Next, we evaluated the effects of C- or G-pre-miR-33 overexpression on permethrin knockdown and mortality phenotypes. To do this, VU mosquitoes were injected with isoform-specific pre-miRNA transcripts 3 days prior to exposure to discriminating doses of permethrin, 1.5 µg per bottle. Endogenous miRNA processing was expected to process the pre-miR-33 hairpin to its mature form. In general, we defined resistance phenotypes as (1) paralytic toxicity, which was monitored in 10 min increments for 1 h, and (2) lethal toxicity, which was assessed at 3 days post-permethrin exposure. Intriguingly, VU mosquitoes with over-expressed G-pre-miR-33 showed increased permethrin knockdown rates compared to the over-expressed C-isoform and non-specific dsRNA injected controls (Fig. 4a, ANOVA **p = 0.0042). In contrast, C-pre-miR-33 treatment groups showed decreased knockdown rates compared to controls. Markedly, by 3 dpt, mortality rates in both G-pre-miR-33 and C-pre-miR-33-treated groups converged to significantly less than that of negative controls (Fig. 4b, Fisher’s Exact test, ***p = 0.0002 and ***p = 1.0 × 10^–5^, respectively). Depletion of miR-33 partially restored high mortality rates (Fig. 4b). VR mosquitoes showed a different pattern, possibly due to the requirement of much higher doses of permethrin in bottle assays (16 µg per bottle). In VR, isoform-specific differences in knockdown rates were not apparent (Supplementary Fig. S3d). By 3 dpt, mortality rates in G-pre-miR-33 treated groups were lower than that of negative controls (Supplementary Fig. S3e, right Y axis, Fisher’s Exact test, *p = 0.02) but less marked than were observed in VU. The results led us to conclude that miR-33 regulates expression of the major permethrin target, VGSC, and that pre-miRNA processing may differ between the two mosquito lines.

### Deep sequencing reveals multi-functional gene sets

To investigate permethrin-associated polymorphisms that arose among VU and VR after about 24 generations in reproductive isolation, target-capture gDNA high throughput sequencing was performed, and SNPs were identified^26,31,32,40^. The goal was to implicate genes genetically associated with the release from selection for permethrin resistance. From 42.7 million to 56.3 million 150 nt reads were produced per dual replicate library of pooled mosquitoes (n = 22 adult females per pool, Supplementary Table S4). SNPs were expected to arise from genetic drift or lack of permethrin selection over time. SNP frequencies were compared between resistant and susceptible mosquito pools using contingency χ^2^ analysis. The − log_10_ (χ^2^ *p* value) was calculated at each SNP site, and final gene-wise weighted averages were determined (see “Methods”).

Genes with weighted average − log_10_ (χ^2^ *p* value) ≥ 15 (n = 2112) were identified, one of which was a long non-coding RNA (AAEL026905, location chr 3: 348,887,920–348,889,837). Five hundred forty nine, 573 and 990 association genes were located on chromosomes (chr) 1, 2 and 3, respectively (Fig. 4c). Overall, the gene set comprised multiple predicted functional categories, from transport to signal transduction and metabolism to cytoskeleton/structural (Supplementary Table S3). In our previous study, we identified genes with predicted associations with motoneuronal function^26^, including the permethrin target, VGSC. In the current work, twenty-three neuronal-associated genes, including VSGC, were present in the highlighted gene set (Fig. 4c, Supplementary Table S3). This is consistent with previous reports that selection at motoneuronal genes is a distinguishing difference between permethrin-resistant and susceptible populations^26,32^. There were 64 genes with predicted involvement in detoxification and/or oxidation/reduction. Of these, 16 CYP orthologs were present. Nineteen of the 2112 genes were predicted to be involved in RNA processing. Moreover, 41 genes were predicted protein translation components.

In our previous genetic association studies, VGSC had the highest genetic association values^26,32^, which is not surprising since it is the target of pyrethroids. Here, VGSC was among the top 14% of values. Notably, there were 292 genome-wide and 147 chr 3 genes with higher − log_10_ (χ^2^ *p* value) than VGSC (Fig. 4c). Predicted functional groups from a wide variety of cellular pathways were represented in this subset (Fig. 4d). Six metabolic resistance components in the genome-wide subset with values higher than VGSC included CYP orthologs (CYP6AG3, CYP6AG4, CYP6AG6, CYP6AG7, CYP12F8, and one mitochondrial CYP) and one peroxidase (Supplementary Table S3).

Gene set enrichment analysis was performed; the functional category of transport was over-represented compared to the proportion of genes in the predicted probe set (hypergeometric distribution, p = 2.86 × 10^–4^). Metabolic genes showed a trend for over-representation but did not pass the multiple testing adjustment cut-off (p = 0.0233, Bonferroni cut-off 0.007). The category of DNA repair/replication/transcription/translation (RRTT) was not significantly over-represented, though it was the most represented among specific functional categories with higher genetic association values than VGSC. Nevertheless, the phenotypic data highlighted in Figs. 1, 2 and 3 were consistent with the involvement of the RRTT category in permethrin resistance.

## Discussion

Permethrin binds to VGSC in susceptible mosquitoes, resulting in neuronal depolarization, disruption of motoneuronal synapses, with possible damage to VGSC protein complexes^12^. Toxic effects, such as paralysis or knockdown, may occur concomitant with stimulation of VGSC transcription^38^ and translation as the mosquito’s system attempts to replenish cellular polarity required for motoneuronal function (Fig. 4), while simultaneously detoxifying and excreting permethrin metabolic intermediates^19^. Investigation of two genetically related *Ae. aegypti* strains revealed differences in the molecular regulation of permethrin resistance. The original Vergel collection (F_3_) had an LC_50_ of about 25 µg permethrin^37^. Importantly, the high level of resistance was selected under natural conditions; it was then maintained for mosquitoes in colony by periodic selection for permethrin resistance (VR strain). In contrast, the permethrin-susceptible strain, VU, lost resistance over 24–27 generations.

In susceptible individuals, such as VU, with higher basal VGSC transcript levels (Fig. 3d), concomitant G-pre-miR-33 over-expression and permethrin exposure appeared to exacerbate paralysis (Fig. 4a). Reasons for increased permethrin-induced knockdown upon G-pre-miR-33 over-expression over that of C-pre-miR-33 are not understood. We speculate that excess miR-33 and subsequent VGSC transcript depletion could have delayed expression of sufficient VGSC protein levels for reactivation of motoneurons. The following study provided support for this idea. Mee et al. described activity-dependent feedback of VGSC (PARA) transcript expression in *Drosophila* embryos^38^, such that, in the absence of synaptic activity, as would occur as part of the permethrin toxicological response, VGSC transcript expression increased, whereas expression subsided when synaptic excitatory levels were high. This nuanced feedback mechanism was not measured in our assays.

**Figure 5:**
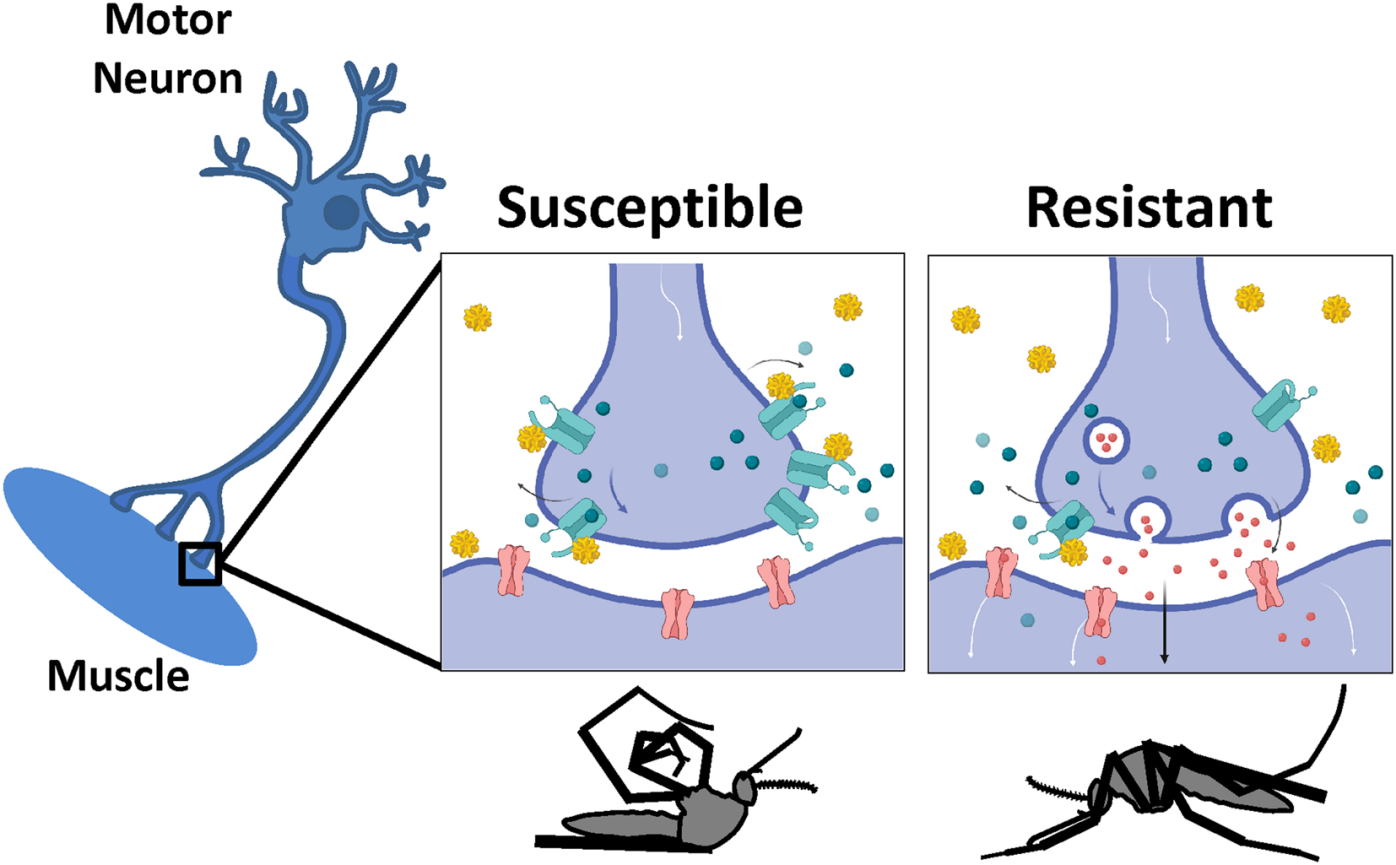
Model depicts differential responses to permethrin treatment in motoneurons of susceptible and resistant mosquito populations. Susceptible mosquito motoneurons depolarize upon permethrin binding to VGSC, leading to cessation of neurotransmitter release, paralysis and possibly death (left panel). Vergel resistant mosquitoes, with reduced VGSC transcript and protein levels, have fewer permethrin binding sites on motoneurons, and therefore less depolarization and subsequent toxicity. Permethrin molecules are shown in bright yellow spheres. Aqua molecules depict VGSC. Red, aqua and blue circles depict neurotransmitters. Pink receptors depict neurotransmitter receptors. Images drawn in Biorender (Biorender.com), with permission.

Reduced VGSC expression is a notable feature of the VR strain. In particular, VR showed lower VGSC protein and transcript levels than did VU (Fig. 3c,d), which would be expected to coincide with fewer permethrin binding sites in VR, and coupled with selection at metabolic resistance genes, would subsequently lead to lowered likelihood of fatal toxicity upon permethrin treatment. Specifically, if fewer VGSC protein complexes were present in motoneurons (Fig. 5), the effects of permethrin toxicity would be much less severe due to the presence of fewer permethrin-target binding events. Metabolic resistance effectors could then detoxify and subsequently clear the insecticide over time.

Study of *Drosophila* DDT-resistant and susceptible strains revealed roles for miRNAs in regulation of metabolic resistance components^41^. Though miR-33 was catalogued in the Seong et al. report, CYP, glutathione S-transferase and cuticular protein gene expression was controlled largely by members of the miR-310 gene cluster (e.g., miR-311-3p, miR-312-3p, miR-313-3p), miR4919-3p, miR-286-3p and others. Nevertheless, the possibility remains that miR-33 targets other than VGSC could aid recovery from permethrin knockdown, and thereby contribute to the phenotypes depicted in Fig. 4a. To explore this possibility, we examined data reported by Wessels et al.^30^, which described AGO1-immunoprecipitated miR-33 targets in *Drosophila* S2 cell culture, a macrophage-like line derived from embryonic cells. No pyrethroid metabolic resistance genes, such as CYPs, mixed function oxidases or Redox components were identified as miR-33 targets. However, of 209 miR-33 targets identified in Wessel’s immunoprecipitation/sequencing screen, 3 known stress response genes were identified, specifically AKT^42,43^, HANGOVER^44^, and CERT^45^ (ceramide transfer protein). Moreover, 12 miRNA targets were synaptic or neural function components (AKT, CERT, SPN (spinofilin), PROSAP, aPKC (atypical protein kinase C), DIKAR, S6KII (ribosomal protein S6 kinase II), PMCA (plasma membrane calcium ATPAse), STATHMIN, TENASCIN, CABEZA, TAO). This is striking, given that the primary toxicological response to permethrin is neurotoxicity at neuromuscular junctions. Together, these data are supportive of a role for miR-33 in nuanced control of neuronal function, rather than metabolic resistance.

VU VGSC protein levels are similar in C-pre-miR-33 overexpressed individuals and βGAL-injected controls (Fig. 3c). In contrast, over-expression of the G-isoform resulted in diminished VGSC levels. Nuclease mapping results were consistent with destabilization or increased breathing of the G-pre-miR-33 hairpin, as shown by more susceptibility to RNAse A treatment in the presence of physiological salt concentrations (150 mM) (Fig. 1a). The breathing propensity of the top section of G-pre-miRNA is apparently not found in C-pre-miRNA. Three bases (U46, C43 and C39) in C-pre-miRNA do not appear to be very single stranded as illustrated by not being highly susceptible to RNAse A cleavage. Some this could be caused by altered base-pairing of C43 and U46 (see dotted line), due to N3–N3 or 2-carbonyl-amino interactions. The less stable structure of G-pre-miR-33 may facilitate processing by DICER-1. Conversely, C-pre-miR-33 showed resistance to nuclease cleavage and an overall more based-paired nature, which could result in hampered DICER-1 accessibility, and thus relatively slower pre-miRNA processing. Functionally, the more fixed structural orientation at the top of C-pre-miRNA could (1) attract RNA binding proteins that interfere with DICER-1 processing or (2) force a specific structural conformation that slowed processivity by DICER-1. In vivo, heterozygous mosquitoes may show variable expression of C- and G-pre-miR-33 isoforms to condition permethrin knockdown rates by providing subtle regulatory changes to downstream mRNA targets, such as VGSC.

We hypothesized that the increased stability of the C-pre-miR-33 isoform would lead to slower processing than occurred for the G-isoform, which would subsequently slow regulation of VGSC levels. Specifically, the breathable G-pre-miR-33 configuration may have decreased the half-life of the pre-miRNA, which could have led to reduced miRNA levels and subsequently reduced target regulation. This result is consistent with increased permethrin-induced mosquito knockdown in the presence of miR-33 inhibitor. Nevertheless, within 3 days, mosquitoes recovered from paralysis and showed increased survival compared to controls (Fig. 4b), possibly due to miR-33-induced reduction of VGSC transcript levels or unknown resistance targets. Consistent with this observation, G-pre-miR-33 overexpression resulted in depleted VGSC transcript levels (Fig. 3b). Taken together, these data suggest a model in which miR-33 regulates VGSC transcript levels.

VGSC protein levels were not completely ablated in either VR miR-33 over-expression group. This could be due to (1) synaptic activity dependent feedback control of VGSC expression^38^ that might self-stimulate transcription and translation, indicating that basal expression is essential, (2) an indirect effect, e.g., miR-33 may regulate a gene upstream of VGSC, or (3) sRNA injections did not completely permeate all neuronal tissues in vivo. Alternatively, the resistant strain may have lost its sensitivity to the effects of miR-33 G- and C-allele differences. Indeed, higher constitutive miR-33 expression in VR over VU is consistent with this interpretation (Supplementary Fig. S3c).

Examination of deep sequencing data revealed genes in a variety of functional groups that were significantly different between VR and VU (Supplementary Table S3). We identified 1 non-coding and 2111 coding genes with average − log_10_ (χ^2^ *p* value) levels above 15, indicative of multi-gene selection following release from permethrin selection. The genetic association data indicated that multiple cellular processes were affected in the resistant population, and these effects could have fitness costs. For example, after 26 generations, 65% of VU individuals reverted to homozygous susceptible alleles, supporting the idea that fitness costs are associated with KDR. Nevertheless, it’s important to note that some genes in the genetic association set could be present due to genetic drift that occurred over time in colony.

There have been limited studies describing the extent to which miRNAi regulates pyrethroid resistance in mosquitoes and other dipterans. To date, a few reports have described miRNAi regulation of the expression of CYPs and other metabolic resistance effector genes^41,46,47^. The current work is the first report of post-transcriptional regulation of VGSC expression. Therefore, at this time it cannot be ascertained whether the features of VGSC regulation described herein are common to pyrethroid resistant populations or whether they merely represent features specific to the Vergel strain. Therefore, it is possible that altered regulation of VGSC expression may have arisen in the Vergel strain through genetic drift or local selection. Clearly, coordinated analyses of miR-33 and VGSC levels in natural mosquito populations will have to be done in order to determine the range of this newly identified regulatory feature of resistance.

Inherited contribution of the KDR genotype, selection at metabolic resistance genes and elevated basal miR-33 levels in VR may work together to condition resistance. A portion of the difference between VU and VR phenotypes could be attributed to the differences in the amount of permethrin required for knockdown of each of these strains. While a 1.5 μg discriminating dose was required for VU, a 16 μg dose was required to obtain a similar effect in VR (Supplementary Fig. S3d,e). Notably, the miR-33 polymorphism, per se, did not appear to contribute to the phenotypic differences between VR and VU. Rather, our data suggest that alterations in mRNA or miRNAi processing occur in VR, consistent with a lack of difference in mature C- and G-miR-33 levels following over-expression, in contrast to those of VU. We speculate that changes in RNA and/or miRNA processing may be a way to compensate for the fitness costs associated with coding mutations associated with resistance (Fig. 2, Supplementary Table S3)^48^. Moreover, within the high association gene set, the presence of multiple genes with predicted association with RNA processing is consistent with the idea that RNA processing is altered in permethrin resistant mosquitoes. Specifically, post-translational, epistatic or other changes could have affected processivity of RNA processing gene products to condition resistance to knockdown.

Post-transcriptional regulation of gene expression could be manifested in multiple ways to enhance insecticide resistance. Stress-responsive post-transcriptional RNA regulatory processes include altered polyadenylation^49,50^, cap-independent internal ribosome entry^51^, and tRNA processing^52^. Components from all these systems were present in our high association dataset. These processes are perturbed during stress in a variety of organismal systems^53,54^. Due to strong evolutionary conservation of these processes, we speculate that part of the mosquito stress response to permethrin exposure selects for altered post-transcriptional regulation of metabolic and knockdown resistance effectors, in addition to selection and/or duplication of metabolic resistance genes.

## Conclusion

The resistant strain, VR, had markedly reduced basal VGSC transcript and protein levels compared to the genetically-related susceptible strain, VU. VR also showed less permethrin-associated sensitivity to over-expressed pre-miR-33 isoforms, which, combined with the KDR genotype profile, selection at metabolic resistance genes and lower basal VGSC transcript levels, likely contributed toward a higher knockdown resistance phenotype. In addition, upon release from permethrin selection, the genetically related VU strain showed evidence of selection at multiple loci involved in cellular transport, signal transduction, redox/mitochondrial function and metabolic functions. Together, these results show that selection of permethrin resistance occurs at multiple loci in vivo.

## Methods

### Mosquito rearing and maintenance

The populations used for this work were collected from the Merida region of Yucatan, Mexico. Location and collection specifics are described in detail in Campbell et al. and Saavedra et al.^26,36^. The Vergel collection (VU) has subsequently been reared in the laboratory and permethrin resistance waned over time; generations F_24_—F_27_ were used for the experiments reported here; subsequently, this strain regained substantial sensitivity to pyrethroid treatment, compared to the original collection. A portion of the original Vergel collection was used to generate a resistant population (VR). The mosquito colonies were kept at high numbers (~ 5000 per generation) to prevent genetic bottlenecks introduced by periodic permethrin treatment of VR and reduce effects of genetic drift. Every third generation, the mosquitoes were exposed to 10–25 µg permethrin (Sigma-Aldrich cat #45614); all those mosquitoes that were unaffected by or recovered from permethrin treatment, typically 30–70%, were used to generate eggs for the subsequent generation. Generations F_26_ and F_27_ were used for experiments described here.

Mosquitoes were hatched from eggs in sterile water and fed on yeast and 10% liver powder until pupation. Adults were held in an environmental chamber at 80% humidity and 28 °C and fed 10% sucrose ad libitum. Adult females were used for all experiments, unless otherwise indicated.

### Genome analyses of published sequencing libraries

Previously published deep sequencing libraries^26^ were analyzed to identify the coverage of all miRNAs. All interrogated sequencing data are available at the National Center for Biotechnology Information (NCBI) Sequence Read Archive, Bioproject accession number PRJNA393171. VCPA permethrin-treated resistant replicates 1 and 2 are SRR5805471 and SRR5805472, respectively. VCPD permethrin-treated susceptible replicates 1 and 2 are SRR5805473 and SRR5805470, respectively. VePA permethrin-treated resistant replicates 1 and 2 are SRR5805465 and SRR5805464, respectively. VePD permethrin-treated susceptible replicates 1 and 2 are SRR5805475 and SRR5805474, respectively.

All predicted pre-miRNAs from miRBase.org were blasted (ncbi-blast-2.2.28 + stand-alone package, blastn) against the AegL5 genome to identify their genome intervals. Next, trimmed fastq files were aligned to AegL5 reference genome using GSNAP, as described below under sequencing. The resulting .bam files were interrogated to identify intervals corresponding to the predicted pre-miRNAs, and SAMtools depth command was used to identify the nucleotide coverage at each site. R scripts were used to extract the coverage of each pre-miRNA interval; the coverage was then graphed using Prism GraphPad version 8.0.1 (GraphPad Software, Inc.).

### Permethrin bottle bioassays and LC_50_ analyses

At 3 days-post–pre-miRNA injection, pools of mosquitoes were subjected to standard CDC bottle assays^55^ using the discriminating doses of 1.5 µg permethrin (Sigma-Aldrich cat #45614, 58.5% trans-isomers, 39.3% cis-isomers) for the VU strain and 16 µg for VR. Knockdown was measured in 10-min increments for one hour, and mortality was scored at 72 h post-treatment. Knockdown data represent a compilation of three experimental replicates. LC_50_ protocols followed those previously described by Saavedra et al*.*^56^; briefly, LC_50_ values were determined by testing 20 mosquitoes per bottle with 3 biological replicates at five doses for each population and monitoring of mosquito activity rates every 10 min for 1 h and mortality at 24 h post-treatment.

### *VGSC KDR* allele frequency analysis

Primer sequences for all genotyping analyses are in Supplementary Table S5. Allele-specific PCR to detect individual V1016I genotypes by melting curve analysis^56^. Each reaction contained 50 μM of two forward primers V1016fw, I1016fw and 50 μM of reverse primer 1016rev, 10 μl Sybr Green Master mix (BioRad, Hercules CA), 9.7 μl ddH20 and 1 μl of genomic DNA (~ 25 ng). PCR and melting curve analysis were run in a CFX-96 (BioRad) following 3 min at 95 °C, 39 cycles of 10 s at 95 °C, 10 s at 60 °C, 30 s at 72 °C followed by a melting curve from 65 to 95 °C with increments of 0.2 °C during 10 s. The products consisted of a 99 bp amplicon for the susceptible allele V1016 and a 79 bp amplicon for the resistant allele I1016.

Individual F1534C genotypes were also detected by melting curve analysis^57^. Each reaction contained 0.5 μM of forward primer F1534fw, 0.165 μM of C1534fw and 0.5 μM of reverse primer 1534rev, 10 μl Sybr Green Master mix (BioRad, Hercules CA), 9.53 μl ddH20 and 1 μl of genomic DNA (~ 25 ng). PCR and melting curve analysis were run in a CFX-96 (BioRad) following 3 min at 95 °C, 39 cycles of 10 s at 95 °C, 10 s at 57 °C, 30 s at 72 °C followed by a melting curve from 65 to 95 °C with increments of 0.5 °C over 5 s. The products consisted of a 113 bp amplicon for the mutant allele C1534 and a 93 bp amplicon for the susceptible allele F1534. Finally, V410L allele frequencies were detected using the methods of Saavedra et al., detailed in^48^.

### Pre-miR-33 gene sequencing and genotyping

We used an allele-specific PCR system to detect individual miR-33 genotypes, using primers listed in in Supplementary Table S3. PCR and melting curve analysis were run in a CFX-96 (BioRad) following 3 min at 95 °C, 39 cycles of 10 s at 95 °C, 10 s at 60 °C, 30 s at 72 °C followed by a melting curve from 65 to 95 °C with increments of 0.5 °C, over 5 s. Twenty mosquitoes per group were tested (Supplementary Table S2). Five per group were validated by Sanger sequencing (Genewiz).

### Pre-miRNA preparation and injections

Primers for dsRNA preparation, RT-qPCR and constructs for miR-33 over-expression are shown in Supplementary Table S5. Pre-miRNAs were synthesized (Integrated DNA Technologies) and inserted into plasmids. Pre-miRNA transcripts were prepared by cutting the 3′ end of each pre-miRNA plasmid at an EcoRV site and performing in vitro transcription, using standard methods^58^. Mosquitoes were injected (Nanoject II, Drummond) with about 200 ng pre-miRNA for over-expression studies. MiR-33 was depleted by injecting mosquitoes with mir-33 hairpin inhibitor (IH-300509-08-0002, Dharmacon), which was expected to deplete mature miR-33 levels. Depletion was validated by RT-qPCR, as described below. All injected mosquitoes were assayed by RT-qPCR, immunoassay or subjected to permethrin bottle assays at 3 dpi. A portion of the Beta-galactosidase gene (βGAL) was used to generate dsRNA for use in negative controls using methods described previously^58^.

Total RNA was extracted from treated mosquitoes using the MiRVana small RNA extraction kit (Ambion). Reverse transcription (5 ng/µl input RNA) and qPCR of mature miR-33 was performed using the manufacturer’s protocol for the Qiagen miRCURY LNA miRNA RT and SYBR green PCR (Qiagen) and the commercially available hsa-miR-33a-5p miRCURY LNA miRNA PCR Assay (Qiagen), which is identical in sequence to aae-miR-33a-5p. Ct values were normalized to aae-miR-let-7 and those of βGAL-dsRNA injected controls. The comparative Ct quantitation (ΔΔCt) method was used to assess differences between treatment groups^59^. Statistical analysis was performed on all technical (n = 3) and biological (n = 3) replicates in Prism Graphpad. Pool sizes for the total number assayed (over all biological replicates) were as follows for the bottle assays-VU: βGAL-injected, n = 86; G-pre-miR-33-injected, n = 228, C-pre-miR-33-injected n = 103, inhibitor, n = 128; VR: βGAL-injected, n = 96; G-pre-miR-33-injected, n = 79, C-pre-miR-33-injected n = 94, inhibitor, n = 85.

### Target gene quantitation

Mosquitoes were injected with pre-miRNAs or βGAL-dsRNA as indicated and held for 3 days prior to collection for RNA extractions. Total RNA was extracted from pools of 3 or 5 adult mosquitoes (as indicated) or larger mixed sex pools of early life stages (larvae-L1-2, L3, L4, pupae or adult females), using Trizol (Invitrogen) and the manufacturer’s recommendations. Reverse transcription (RT) reactions (QuantiNova, Qiagen) used 3 µg input per reaction. RT reactions were diluted 1:10 prior to quantitative PCR (QuantiNova, Qiagen) on a CFX96 instrument (BioRad); triplicate technical and biological replicates were performed for all samples. ACTIN and RPS7 were used as reference standards for all reactions. Relative gene expression levels were calculated using the ∆∆Ct method, with untreated adult VU females as the calibrator. Relative expression differences were compared using ANOVA in Prism GraphPad.

### Nuclease sensitivity assays

C-pre-miR-33 and G-pre-miR-33 were analyzed using mfold (http://unafold.rna.albany.edu/?q=mfold) to determine provisional secondary conformations (Fig. 1)^34^. RNAs were transcribed using SP6 RNA polymerase from DNA templates (Supplementary Table S5) purchased from IDT that encoded either isoform. RNAs were transcribed in the presence of a 10X concentration of 5′ GMP relative to 5′GTP to obtain transcripts with a 5′ monophosphate. RNAs were treated with calf intestinal phosphatase and 5′ end labeled using T4 polynucleotide kinase (New England Biolabs) and gamma ^32^P-ATP. Radioactively end labeled RNAs were gel purified on a 5% acrylamide gel. An equal amount of radioactive counts of each RNA (which also represents an equimolar amount of each transcript) was incubated at room temperature with 1 µg/ml RNAse A for 5 min in 150 mM NaCl, 50 mM Tris–HCL (pH7.9), 10 mM MgCl_2_ and 1 mM DTT. Reaction products were purified by phenol–chloroform extraction, concentrated by ethanol precipitation, separated on a 5% acrylamide gel containing 7 M urea, and analyzed by phosphorimaging. Nucleotide positions were assigned by aligning bands using an RNA ladder as well as RNA partially digested using T1 RNAse to identify the positions of G residues. Computer-aided RNA structural models were prepared using the mfold server.

### Immunoblots

We extracted protein from pools of five mosquitoes using 200 µl of radioimmunoprecipitation assay (RIPA) buffer 50 mM Tris HCl pH 8, 150 mM NaCl, 1% NP-40, 0.5% sodium deoxycholate, 0.1% SDS) and 2 µl of a 100X Halt protease inhibitor cocktail (ThermoScientific). Mosquitoes were ground with a hand-held tissue homogenizer (VWR) with plastic pestles for a minimum of 30 s. Homogenates were centrifuged at maximum gravity at 4 °C for 15 min. We proceeded immediately to protein quantitation using reagents from the Better Bradford assay (Pierce). Equivalent amounts of protein were loaded onto 10% SDS polyacrylamide gels (TGX, BioRad) and ran at 150 V until the leading band approached the bottom of the gel. The protein was transferred to PVDF membranes (Novex Invitrolon) at a constant current of 350 mA for one hour at 4 °C. Blots were blocked in a 5% non-fat milk blocking solution prepared in 1X TBS-T. All primary antibody dilutions were prepared in 5% non-fat milk in TBS-T. All secondary antibody dilutions were prepared in TBS-T. Antibodies: rabbit anti-pan-VGSC primary antibody (Millipore AB5210) at 1:200, rabbit anti-β-ACTIN primary antibody (Sigma A5060) at 1:250, goat anti-rabbit secondary antibody-HRP (Invitrogen, REF#31460) at 1:10,000, donkey anti-goat secondary antibody (Abcam ab6885) at 1:10,000. ELC chemiluminescent kit (Thermofisher), and the manufacturer’s recommendations were used to image blots on a ChemiDoc (BioRad), using the Chemi Hi Resolution setting.

### Deep sequencing of VR and VU

Genomic DNA libraries (PCR-free gDNA preparation kit, Illumina) were prepared for dual replicate pools of 22 mosquitoes each from the VR (2 replicates) and VU colonies (2 replicates). A total of 4 paired-end read libraries were analyzed to identify SNPs of interest. We used the same methods as previously reported^26,32^. Briefly, equimolar quantities of prepared libraries were pooled and enriched for coding sequences by exome capture using custom SeqCap EZ Developer probes (Nimblegen)^40^. Overlapping probes covering protein coding sequences (not including UTRs) were produced by Nimblegen. Our capture probes were developed prior to the full-length assembly of the Aaeg LVP_AGWG L5 genome; the probes cover 77% of the genes (13,942/18,081) in L5^26^; probe coordinates are listed in Supplementary Table S6. Enrichment followed the Nimblegen SeqCap EZ protocol. Briefly, pooled gDNA libraries were hybridized to the probes for 48 h at 60 °C, unbound DNA was washed away, and the targeted DNA was eluted and amplified (12 cycles). These were sequenced on a NovaSeq (Illumina) at University of Colorado for paired-end 2 × 150 nt sequencing, producing reads with quality scores > 30. Briefly, trimmed fastq reads were aligned to AaegL5 reference genome^60^ using GSNAP (version 2019-06-10), allowing 10% divergence^61^. GSNAP outputs were converted to pileup using SAMtools^62^. The “readcounts” command in Varscan2 (v2.3.5)^63^ was used to convert pileup files to readcounts output, using the following options: –min-coverage 22 –min-base-qual 30. The minimum coverage requirement was set to match the number of diploid genomes in each pool. The R scripts used to analyze the data are available on request. The frequencies of single nucleotide polymorphisms (SNPs) were compared between resistant (VR) and susceptible (VU) mosquito pools using a contingency χ^2^ analysis. Between replicate χ^2^ tests (*p* value cutoff 0.10) were also performed to ensure between-replicate inconsistencies were filtered out. The − log_10_ (χ^2^ *p* value) was calculated at each SNP site, and weighted averages were determined from all sites in each gene to determine each gene-wise score. Benjamini–Hochberg multiple-testing adjustment cut-off of 0.001 was applied. Due to the use of colonized mosquitoes rather than field-collections as used in our earlier studies^26,31,32,40^, a large number of very high − log_10_ (χ^2^ *p* value) genetic association scores were produced. Lower *p* values, and thus higher − log_10_ (χ^2^ *p* value) scores likely occurred due to lower between-replicate variance than would occur with an outbred field collections. Therefore, we used a − log_10_ (χ^2^ *p* value) cut-off of 15 for the data presented here.

Gene annotations were obtained from Vectorbase^64^ using BioMart. Functional groups were manually curated using the following categories: ‘chemosensory response’ (CSR); cytoskeletal/structural (CYT/STR); ‘diverse’ (DIV) for genes with multiple or less clearly defined function(s) or and ‘unknown’ (UNK) for those that are uncharacterized; genes with predicted function in oxidation/reduction processes were categorized according to subsets e.g., (Detox, Detox/Redox, ReDox); those localized to mitochondrial compartment (MIT), regardless of function; proteolysis or proteosomal activity (PROT); replication/(DNA)repair/transcription/translation (RRTT); RNA processing (RNA_PROC) genes were a subset of RRTT that was interrogated separately; signal transduction (SIGT); metabolism or lipid-associated (MET/LIPID). Finally, the ‘transport’ (TRP) category included all gene products predicted to be involved in transport, e.g., moving molecules across membranes, including receptors, exclusive of secondary messengers and signaling receptors.

### Gene set enrichment analysis (GSEA)

One-tailed cumulative hypergeometric distributions of predicted coding genes in each functional category (TRP, MET/LIPID, SigT, Redox/MIT, RRTT, CYT/STR), was performed in R to identify functional categories that were over-represented in our data set. A Bonferroni multiple testing adjustment of p = 0.007 was applied. Of ~ 13,942 genes covered by capture probes, 10,447 had sufficient coverage to pass all filters (− log_10_ (χ^2^ *p* value ≥ 2.98). Numbers of genes in each functional category were compared to the same category in genes represented in the gene set. Parts of Fig. 5 were made in Biorender (https://biorender.com/); used with permission.

### Consent to publish

All authors have given their consent to publish.

## Supplementary Information

Supplementary Information.

Supplementary Tables.

## Data Availability

All sequencing data for VR and VU exome capture trimmed and paired gDNA fastq files are available at National Center for Biotechnology Information BioProject PRJNA594948. Biosample accession numbers are as follows: SAMN13501903, Vergel_Res1; SAMN13501904, Vergel_Res2; SAMN13501905, Vergel_Susc1; SAMN13501906, Vergel_Susc2. The remainder of the data is presented in the main figures and “Supplementary files”.
