# Supplementary material for: Aedes aegypti miRNA-33 modulates permethrin induced toxicity by regulating VGSC transcripts
Source: Sci Rep. 2021 Mar 31;11:7301. doi: 10.1038/s41598-021-86665-6 (PMC8012613; doi:10.1038/s41598-021-86665-6)
Supplement: Supplementary file 1 — Supplementary Information. [file 41598_2021_86665_MOESM1_ESM.docx]

Supplementary information.

**Aedes aegypti** **miRNA-33 modulates permethrin-induced toxicity by regulating** **VGSC transcripts**

**Authors-** Tristan D. Kubik, Trey K. Snell, Karla Saavedra-Rodriguez, Jeffrey Wilusz, John R. Anderson, Saul Lozano-Fuentes**,** William C. Black IV, Corey L. Campbell.


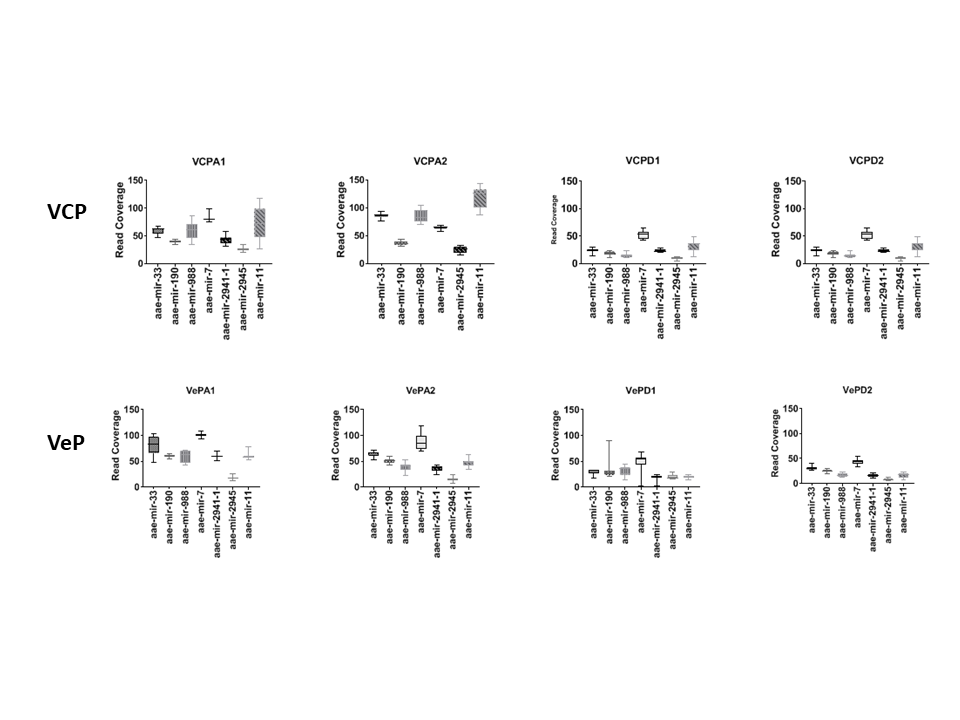


**Supplementary Figure S1.** Previously published sequencing libraries (Campbell et al., 2019; Saavedra-Rodriguez et al., 2019) were analyzed for the presence of miRNAs at coverage levels suitable for association mapping (> 15 reads per nucleotide). Each collection-treatment group (Viva Caucel-permethrin (VCP) and Vergel-permethrin (VeP)), was sequenced in duplicate for each phenotypic category: resistant (xxxA1 and xxxA2) and susceptible (xxxD1 and xxxD2). To determine the average coverage per nucleotide over the interval spanning each pre-miRNA, the samtools depth command was used to interrogate .bam files of trimmed fastq files aligned to the AegL5 reference sequence. Bars indicate minimum and maximum coverage values at each nucleotide position; boxes indicate the 25^th^ and 75^th^ percentiles.

**
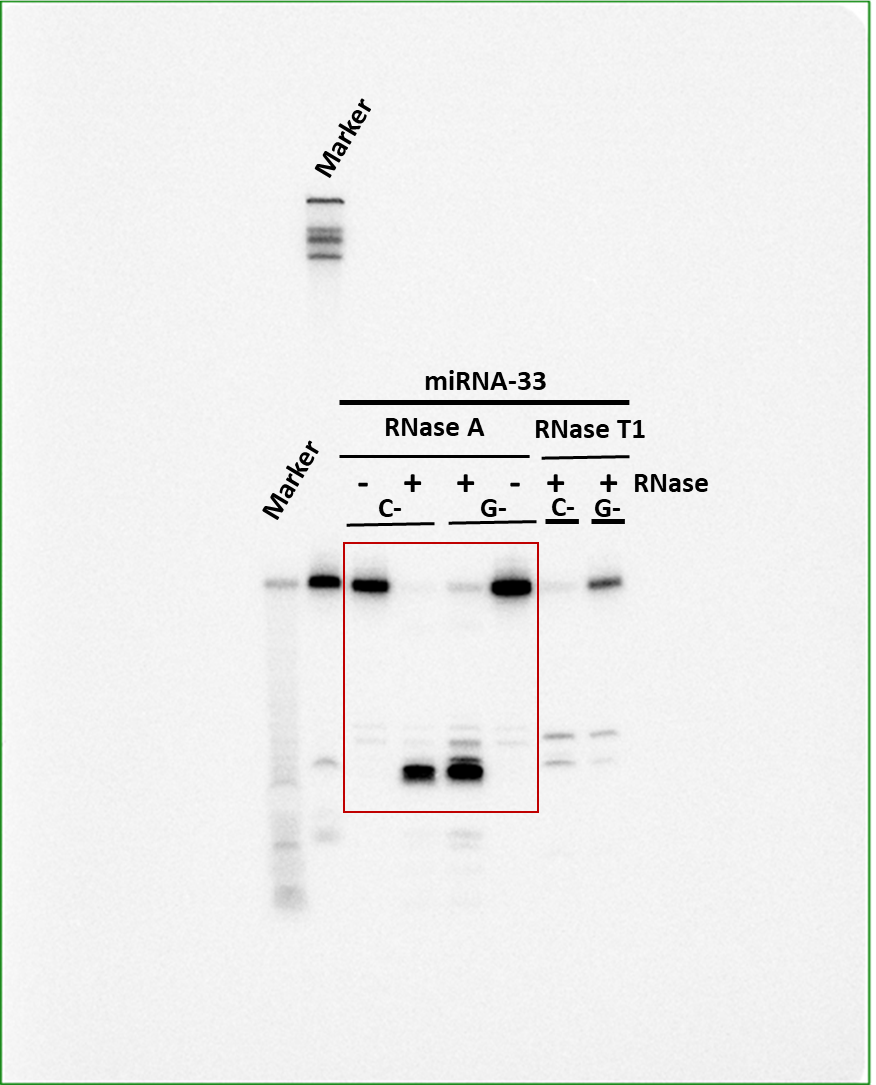
**

**Supplementary Figure S2.** Raw blots of nuclease sensitivity analysis of miR-33 isoforms shown in Fig. 1. The C-pre-miR-33 and G-pre-miR-33 were treated with either RNAse A (RNAse A lanes) or RNAse T1 (RNAse T1 lanes) and analyzed on a 5% polyacrylamide gel containing 7M urea. Lanes with RNAs for sizing and gel orientation (marker lanes) were included on the left.  Nucleotide positions were assigned using an RNA ladder as well as RNA partially digested using T1 RNAse to identify the positions of G residues. Data are representative of at least 3 technical replicates.

**
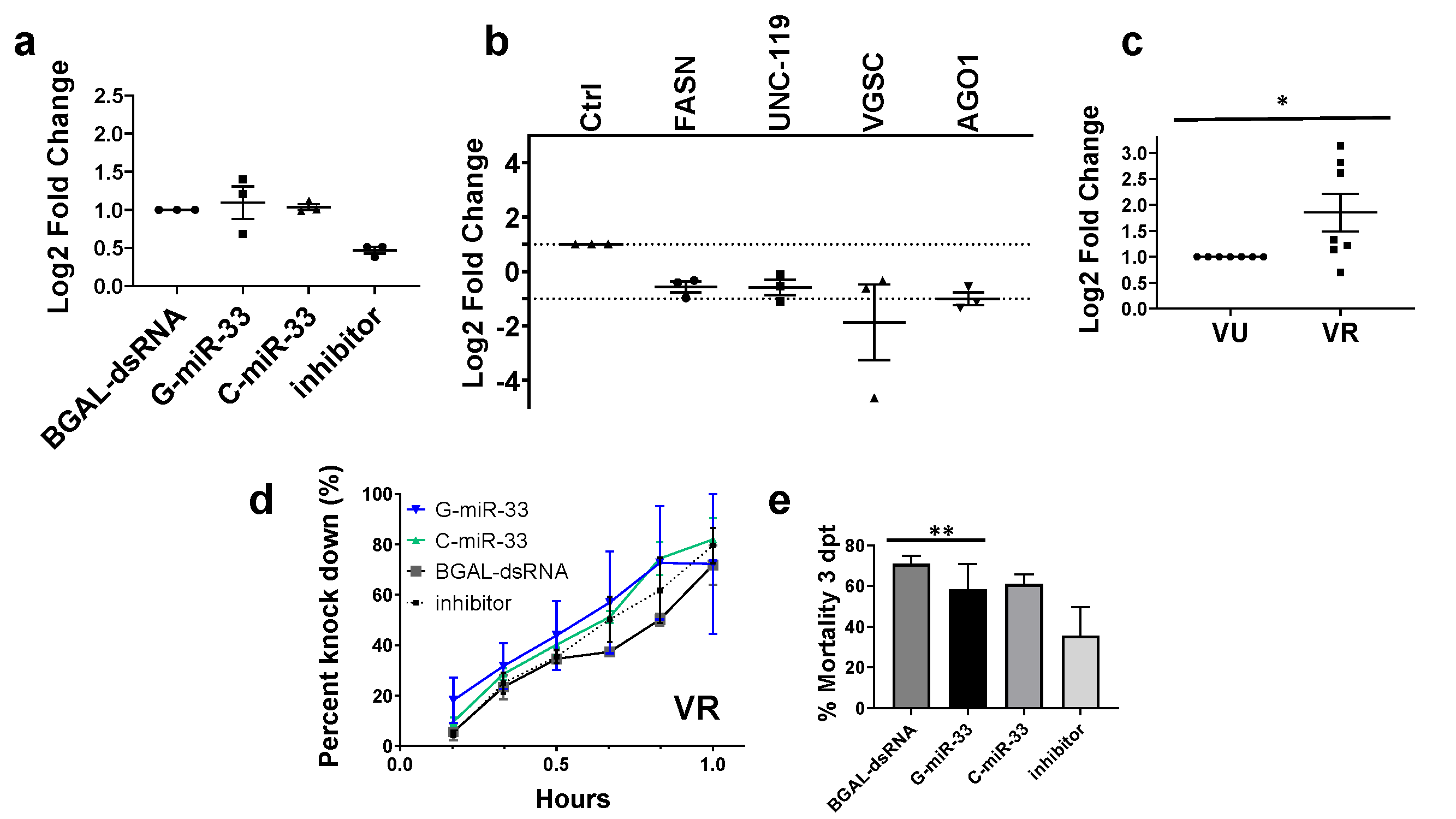
Supplementary Figure S3.** miR-33 pre-miRNA over-expression in VR strain. **(a)** VR strain mature miR-33 levels following over-expression of G- and C- isoforms or inhibitor. **(b)** Decay-sensitive transcripts resulting from G-pre-miR-33 over-expression in VR strain (ANOVA, *p=0.015), relative to ACTIN and RPS7 reference standards and βGAL-dsRNA injected controls (Ctrl). Targets- FASN- fatty acid synthase, UNC-119 GMP phosphodiesterase, VGSC- voltage-gated sodium channel, AGO1- member of the miRISC. Error bars indicate mean with standard error of the mean (SEM). **(c)** MiR-33 levels compared between βGAL-injected VR and VU adult mosquito pools (n=5 per pool, 7 pools). Let-7 miRNA levels were used as reference standards. Error bars indicate standard error of the mean (SEM); data are a compilation of 7 experimental replicates (Mann-Whitney test, *p= 0.0169). **(d)**  Observed permethrin-induced knockdown following C- and G-pre-miR-33s over-expression in adults, using standard bottle assays with 16 µg permethrin at 3 days post-injection (dpi). **(e)** G-pre-miR-33 over-expression decreased permethrin-induced mortality rates (Fisher’s Exact test, **p=0.0029). Data represent the compilation of 3 to 4 experimental replicates


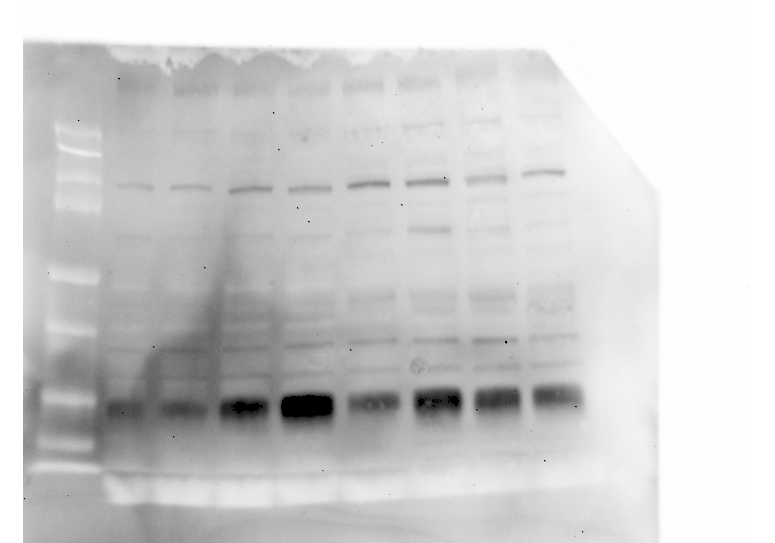
**
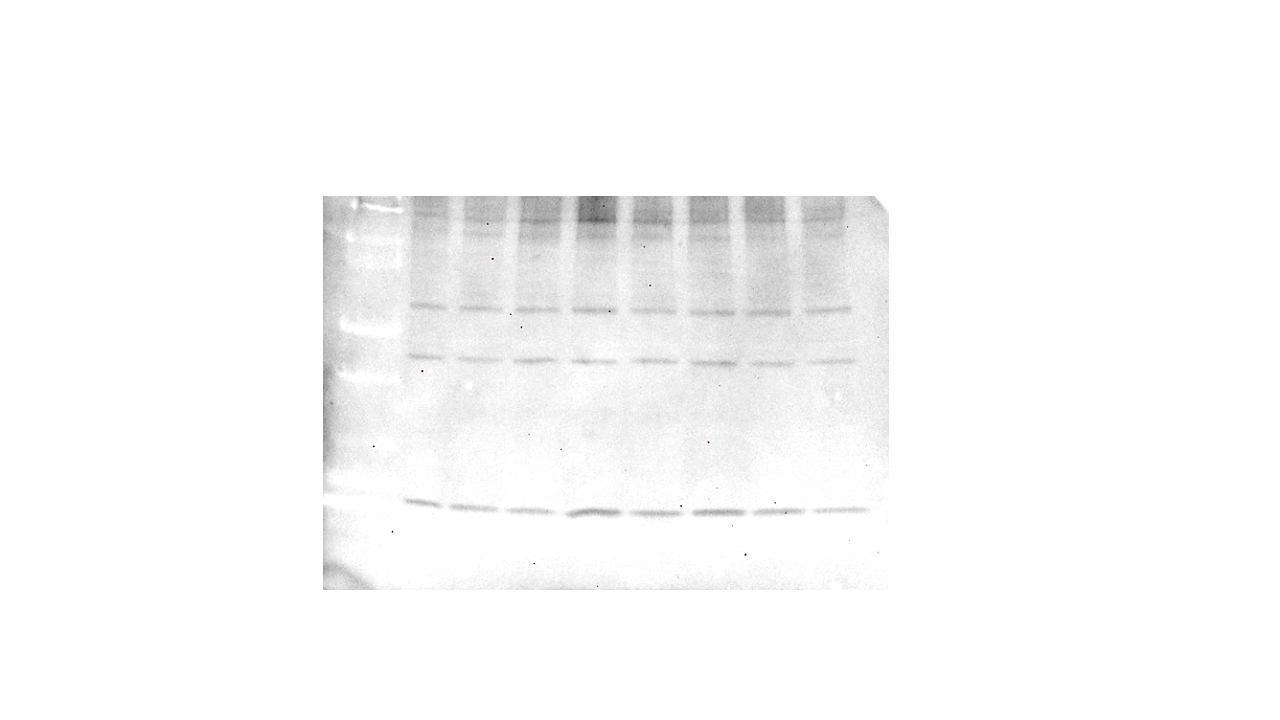
Actin VGSC**

*β*GAL C- G- inhib *β*GAL C- G- inhib

*β*GAL C- G- inhib *β*GAL C- G- inhib

Vergel-resistant Vergel-unselected

Vergel-resistant Vergel-unselected

**Supplementary Figure S4.** Raw immunoblot images. Left panel actin. Right panel, VGSC. Samples are from left to right: Vergel-resistant (βGAL, C-, G-, inhib), Vergel-unselected (βGAL, C-, G-, inhib). Images shown in Fig 2c are indicated with red boxes.

**Supplementary Tables.**

**Supplementary Table S1.** MiRNA SNP locations for the 7 miRNAs assessed for this work.

**Supplementary Table S2.** Mir-33 allele frequency and Sanger sequencing analysis. VU (Vergel-unselected) and VR (Vergel-resistant) do not have significantly different G- and C-pre-miR-33 genotypes.

**Supplementary Table S3.** Highlighted gene set from VR vs VU genetic association mapping. Exome-capture deep sequencing libraries were subjected to SNP-wise contingency analysis. A set of 2112 genes were highlighted (χ^2^ contingency ^–^log_10_(p value) scores greater than 15).

**Supplementary Table S4.** VR vs VU deep sequencing details. Number of reads aligned to reference, % reads mapped, number of variant sites per chromosome, ratio aligned nucleotides*1000.

**Supplementary Table S5.** Primers and pre-miR-33 sequences used in this study.

**Supplementary Table S6.** AaegL5 genome coordinates that correspond to capture probes used in this study.
